# A rare case of intestinal obstruction caused by Meckel's diverticulum band

**DOI:** 10.1016/j.amsu.2022.103807

**Published:** 2022-05-18

**Authors:** Abdullah Alzarea, Alaa Aljohani, Hanan Qabani, Ali Alzahrani, Rami Sairafi

**Affiliations:** General Surgery Department, Security Force Hospital, Riyadh, Saudi Arabia

**Keywords:** Intestinal obstruction, Meckel's diverticulum

## Abstract

**Introduction:**

and Importance: Meckel's diverticulum band is an uncommon cause of intestinal obstruction in adults.

**Case presentation:**

We report the case of a 57-year-old diabetic and hypertensive male who presented with sudden onset of vomiting and abdominal pain. Initial laboratory and imaging investigations, including an abdominal X-ray and computed tomography scan, were suggestive of an intestinal obstruction; however, these were not helpful in assigning a presumptive cause. A laparoscopic exploration was offered after a repeated abdominal X-ray showed an air-fluid level. Intraoperative findings revealed an ischemic closed loop obstructed by a fibrous band of Meckel's diverticulum. Resection and anastomosis of the bowel was performed, and the patient was discharged on day 3 post-operative with no complications.

**Clinical discussion:**

Surgical resection performed by open or laparoscopic approach is the recommended treatment for patients with symptomatic Meckel's diverticulum. Generally, a wedge resection of the Meckel's diverticulum is performed, and occasionally part of the ileum is resected by end-to-end anastomosis, as was the case in our patient.

**Conclusion:**

We believe early surgical intervention is crucial for a favorable outcome. Thus, surgeons should consider complicated Meckel's diverticulum in patients presenting with signs and symptoms of small bowel obstruction.

## Highlights

• Intestinal Obstruction Caused by Meckel's Diverticulum Band are extremely rare in adult.
• Obstruction Caused by Meckel's Diverticulum Band may present a challenging clinical diagnosis.
• Surgical resection remains the treatment of choice and plays a potentially curative role.

## 1 Introduction

Meckel's diverticulum is the most frequent congenital anomaly of the gastrointestinal tract that results from the failure of the vitelline duct to close during fetal development [1]. The condition is usually discovered incidentally during a surgical intervention for another indication and has been reported to cause life-threatening complications, including gastrointestinal bleeding, intestinal obstruction, intussusception, and perforation [2,3]. Amongst these, occlusion of the small intestine has been reported as the most frequent complication in patients with Meckel's diverticulum, representing approximately 36.5% of all complications [4]. The mechanism underlying the development of intestinal occlusion in patients with Meckel's diverticulum includes intussusception of an inverted diverticulum, herniation of a diverticulum, and internal herniation of the small bowel loops underneath the mesodiverticular band [5,6]. We report a case of intestinal obstruction due to Meckel's diverticulum band.

## 2 Case Presentation

This work has been reported in line with the SCARE 2020 criteria [7]. A 57-year-old diabetic and hypertensive man presented to the emergency department with a one-day complaint of abdominal pain and vomiting. He was on oral medications for diabetes mellitus and hypertension, and his surgical history was unremarkable. He reported that the onset of abdominal pain was sudden, and it was mainly localized in the epigastric region. The pain did not radiate to other areas, and it was associated with multiple episodes of vomiting and obstipation. The patient had no history of a similar attack.

On physical examination, he was alert and oriented and his vital signs were stable. His abdomen was soft to palpation, mildly distended and tender, especially in the right lower quadrant. A digital rectal examination revealed hard stools in his rectum. Bowel sounds were heard in the periumbilical area and were normoactive.

**Fig. 1:**
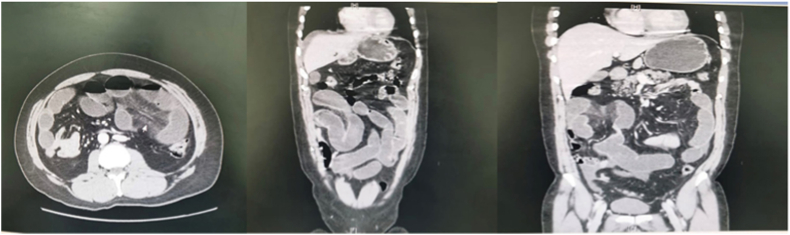
Computed tomography of the abdomen showing high-grade intestinal obstruction with a transition zone.

The results of paraclinical investigations were unremarkable. A complete blood count revealed a white blood cell count of 10 × 10^9^/L and hemoglobin concentration of 15.9 g/L. Lactate and alkaline phosphatase levels were 2.00 mmol/L and 59U/L, respectively. An abdominal X-ray showed multiple air fluid levels and a computed tomography (CT) examination of the abdomen showed a high-grade obstruction with a transition zone within the distal ileum (Fig. 1). There was no sign suggestive of bowel ischemia or perforation.

The patient was admitted to the surgical ward and a nasogastric tube was inserted. Intravenous fluids, prophylactic antibiotics, and analgesia were administered, and abdominal X-rays and abdominal girth measurements were performed daily.

One day after admission, the pain and vomiting resolved but his abdomen was still distended. Additionally, he had no bowel motions or flatus. Gastrographin small bowel follow-through was performed on the second day of admission to assess patient improvement and the effectiveness of conservative treatment, and it showed high-grade intestinal obstruction. The results of further laboratory investigations were unremarkable. However, an abdominal X-ray showed an air-fluid level prompting us to offer a laparoscopic exploration on the third day of admission.

**Fig. 2:**
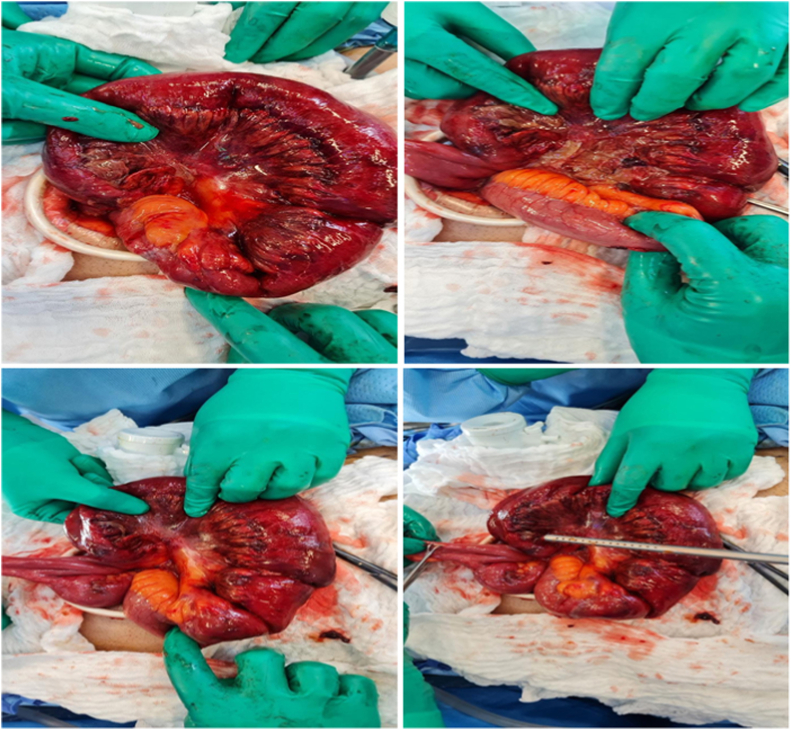
Intraoperative image showing the diverticulum band with an ischemic bowel.

**Fig. 3:**
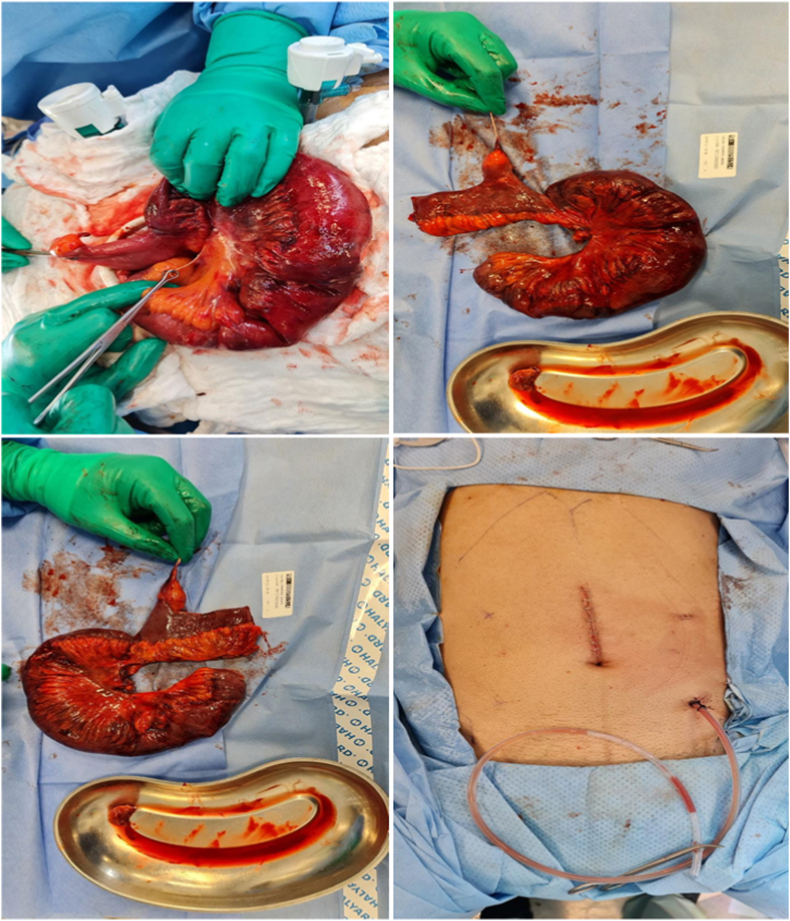
Image showing the resected bowel with the diverticulum band that caused obstruction and ischemia.

Intraoperative findings revealed an ischemic closed loop obstructed by a fibrous band of Meckel's diverticulum (Fig. 2). Resection and anastomosis of the bowel were performed (Fig. 3). The duration of the procedure from anesthesia induction to extubation was approximately 150 minutes. On day 3 post-surgical, the patient was discharged and an examination during follow-up visits was unremarkable. Histopathologic examination identified Meckel diverticulum with heterotopic pancreatic tissue.

## 3 Discussion

Most patients with Meckel's diverticulum do not present any symptoms, and the risk of these patients developing complications is approximately 4–7% [8]. Patients who do develop complications due to Meckel's diverticulum present an unspecific array of symptoms, making diagnosis challenging. In complicated cases, the clinical presentation may mimic other disorders of the gastrointestinal tract such as appendicitis, cholecystitis, inflammatory bowel disease, and lower gastrointestinal hemorrhage [4]. Intestinal occlusion is the most common complication, and it can occur by various mechanisms, including intussusception, mesodiverticular band, small bowel strangulation beneath a band protruding from Meckel's diverticulum to the mesentery base, and stricture due to chronic diverticulitis [8]. In our case, intraoperative findings showed an ischemic closed loop obstructed by a fibrous band of Meckel's diverticulum.

The patient in our case reported a sudden onset of abdominal pain and vomiting and initial imaging investigations, including an abdominal X-ray and CT scan, were not helpful in reaching a presumptive diagnosis. While complementary studies have been reported to be of little or no value in reaching a diagnosis, these may be helpful in complicated cases, with CT being the best imaging modality in these patients [9]. A contrast-enhanced CT scan of the abdomen may miss a Meckel's diverticulum, which may be confused with a small bowel loop [9]. There is no evidence that other modalities such as magnetic resonance imaging have a role in establishing the diagnosis in uncomplicated or complicated cases [10].

Surgical resection performed by open or laparoscopic approach is the recommended treatment for patients with symptomatic Meckel's diverticulum. Generally, a wedge resection of the Meckel's diverticulum is performed, and occasionally part of the ileum is resected by end-to-end anastomosis [11], as was the case in our patient. Several factors determine the resection approach in patients with symptomatic Meckel's diverticulum. These include intestinal tissue integrity and the existence and location of ectopic tissue, which are difficult to accurately predict during surgery by palpation and macroscopic examination of the intestine. Nevertheless, when ectopic tissue is present, the surgeon can predict its location based on the height-to-diameter ratio. Diverticula with a height-to-diameter ratio greater than two, typically have ectopic tissue at the body and tip. Conversely, those that have a height-to-diameter ratio less than two have ectopic tissue distributed widely, including at their base [12]. Thus, the surgeon can use the height-to-diameter ratio to categorize Meckel's diverticulum as long or short and determine the best resection approach. Based on these arguments, when surgery is indicated for patients with complicated intestinal obstruction, wedge or segmental resection is recommended [13].

Laparoscopy was performed in our case; however, traditional laparotomy and laparoscopy have similar outcomes in patients with symptomatic Meckel's diverticulum [14]. According to some investigators [15], the postoperative complications as well as reoperation and readmission rates did not differ between patients who had laparoscopy and those who had laparotomy. Based on their finding of a conversion rate of 27.4%, they suggested that surgeons should avoid routine conversion for palpation of the diverticulum or segmental resection of the small intestine without strong intraoperative evidence or surgical complications [15].

## 4 Conclusion

Overall, a Meckel's diverticulum band is an uncommon cause of intestinal obstruction in patients with Meckel's diverticulum. Surgeons may be faced with the challenge of establishing the diagnosis preoperatively due to its unspecific clinical presentation, leading to a delay in diagnosis. Early surgical intervention is crucial, as this may result in a favorable outcome. Thus, clinicians should consider complicated Meckel's diverticulum in patients presenting with signs and symptoms of small bowel obstruction. Laparoscopic intervention is a safe and feasible way to treat complicated Meckel's diverticulum.

## Provenance and peer review

Not commissioned, externally peer-reviewed.

## Ethical approval

This report was approved by the hospital's Institutional Review Board.

## Sources of funding

None.

## Author contribution

Dr Abdullah Alzarea and Dr. Alaa Aljohani wrote the manuscript. Dr. Hanan Qabani collected the data and compiled references. Dr. Ali and Dr. Rami supervised and guided the writing process. All authors read and approved the final manuscript for submission.

## Research registration (for case reports detailing a new surgical technique or new equipment/technology)

Not applicable.

## Guarantor

The Guarantor is the one or more people who accept full responsibility for the work and/or the conduct of the study, had access to the data, and controlled the decision to publish.

## Consent

Written informed consent was obtained from the patient for publication of this case report and accompanying images. A copy of the written consent is available for review by the Editor-in-Chief of this journal on request.

## Declaration of competing interest

The authors declare that they have no conflict of interest.
